# A Regulatory Network of Transcription and Hormones Controls PCD and Autophagy in Grape Seed Abortion

**DOI:** 10.3390/ijms27188353

**Published:** 2026-09-19

**Authors:** Ying Wang, Pengcheng Zhao, Xuxian Xuan, Qiqi Wu, Ruiqiang Chao, Yan Zhong, Haitao Chen, Yuxuan Song, Lianshuang Liang, Leyi He, Hui Zhou, Zhenqiang Xie, Chen Wang

**Affiliations:** 1 College of Horticulture, Nanjing Agricultural University, Nanjing 211800, China; 2023104031@stu.njau.edu.cn (Y.W.); 2021204006@stu.njau.edu.cn (X.X.); wuqqi883246@163.com (Q.W.); chaoruiqiang1026@163.com (R.C.); yzhong@njau.edu.cn (Y.Z.); 2024104025@stu.njau.edu.cn (H.C.); 15149149427@163.com (Y.S.); 2024804282@stu.njau.edu.cn (L.L.); 2025104026@stu.njau.edu.cn (L.H.); 2025804277@stu.njau.edu.cn (H.Z.); 2 Jiangsu Key Laboratory for Horticultural Crop Genetic Improvement, Institute of Pomology, Jiangsu Academy of Agricultural Sciences, Nanjing 210014, China; zhaopengcheng@jsafc.edu.cn

**Keywords:** grape, seed abortion, programmed cell death, vacuolar processing enzyme, metacaspase, autophagy

## Abstract

The seedless trait in grapes primarily arises from pseudo-parthenocarpy, driven by early seed abortion involving programmed cell death (PCD) and autophagy. However, the specific regulatory networks governing these pathways remain poorly defined. To address this, we systematically characterized the PCD-related (vacuolar processing enzyme [VPE] and metacaspase [MC]) and autophagy-related (ATG) gene families in the seedless cultivar ‘Jingkejing’ utilizing comprehensive bioinformatic analyses, spatiotemporal transcriptomic profiling, and transient expression assays. We identified three VPE, six MC, and 33 ATG genes, whose promoters were significantly enriched in hormone-responsive and transcription factor (TF)-binding motifs. Transcriptomic profiling across critical seed abortion stages highlighted *VvγVPE* and *VvMC9* as key differentially expressed genes. Furthermore, protein–protein interaction predictions indicated extensive crosstalk among PCD/ATG proteins, kinases, and ubiquitin system components. Notably, transient expression assays confirmed that two core transcription factors, VvWRKY46 and VvERF71, specifically regulate downstream PCD-related targets, supporting a proposed multi-hormonal and TF-mediated regulatory network during seed abortion. These findings elucidate the molecular mechanisms underlying pseudo-parthenocarpy, providing essential candidate gene resources for the marker-assisted breeding of seedless grapes.

## 1. Introduction

Seedless grapes are highly favored by consumers and occupy a significant position in the fresh grape market due to their ease of consumption and suitability for value-added processing. Consequently, the genetic improvement in seedless traits and the elucidation of their underlying molecular mechanisms have garnered substantial research attention. Embryo abortion is the primary cause of seedlessness in grapes. A comprehensive understanding of the molecular regulatory networks governing seed abortion not only provides a theoretical foundation for seedless grape breeding but also offers practical value for enhancing fruit yield and quality.

Programmed cell death (PCD) and autophagy are interconnected cellular processes that play critical roles in seed abortion. Vacuolar processing enzymes (VPEs), and metacaspases (MCs) serve as core regulators of plant PCD pathways, while autophagy-related (*ATG*) genes execute the autophagic process [1,2,3,4,5]. Both VPEs and MCs belong to the cysteine protease superfamily, yet they act through distinct mechanisms [6,7,8]. VPEs, which belong to the C13 family of cysteine proteases, are primarily localized in plant vacuoles [9]. Although early studies indicated that VPEs primarily process storage proteins by hydrolyzing peptide bonds, subsequent research revealed that VPEs can mediate vacuolar membrane rupture, releasing proteases into the cytoplasm to trigger PCD [1,10,11]. For instance, during barley seed development, VPEs contribute to the degradation of the nucellus and seed coat, directly influencing seed size [12,13]. As members of the C14 family of cysteine proteases, MCs are evolutionarily conserved across plants, fungi, and protists, serving as integral components of PCD pathways [14]. Loss-of-function studies have shown that MC deficiency impairs PCD in the suspensor, leading to developmental arrest [15]. Recent evidence suggests that MCs not only activate autophagy pathways but also participate in the degradation of intracellular components following vacuolar rupture, representing a critical node for crosstalk between PCD and autophagy [16,17,18,19].

Autophagy, mediated by *ATG* genes, is a crucial catabolic pathway that targets damaged proteins and organelles to the vacuole for degradation and recycling, thereby maintaining cellular nutrient and energy homeostasis [20]. Beyond its role in stress responses, autophagy participates in diverse developmental processes, including anther and ovule development [21,22,23,24,25], sugar metabolism regulation [26], phytohormone signaling [27], and seed yield determination [28]. Moreover, the synergistic or antagonistic crosstalk between autophagy and PCD pathways underscores its multifaceted regulatory role in embryo abortion [29,30,31]. For instance, autophagy modulates wheat inflorescence development to regulate seed formation and enhance yield [28]. During maize endosperm development, the significant upregulation of *ATG8*, *ATG1a*, *ATG3*, and *ATG18c* implicates these genes in endosperm maturation and cell death [32]. In rice, tapetal tissues of autophagy mutants display severe developmental defects compared to wild-type plants, indicating that normal tapetum degradation requires autophagic activity [22]. Collectively, these findings underscore the indispensable role of autophagy in PCD and seed development.

While the fundamental functions of the *VPE*, *MC*, and *ATG* gene families have been extensively characterized, previous research has primarily focused on biotic and abiotic stress responses. Regarding seed abortion, current knowledge relies heavily on model plants such as *Arabidopsis* and rice, with limited studies on important horticultural crops such as grapevine. Moreover, the specific molecular mechanisms, interaction networks, and crosstalk linking these gene families with other signaling pathways during grape seed abortion remain poorly understood. In this study, we systematically identified nine PCD-related genes (representing the *VPE* and *MC* families) and 33 autophagy-related genes (*ATG* family) in the grapevine genome, followed by comprehensive bioinformatic analyses. By integrating spatiotemporal expression profiles with promoter *cis*-regulatory element analysis, we explored their potential roles in underlying seed abortion of seedless grapes. Furthermore, the expression responses of candidate genes to various signaling stimuli were validated using transient transformation in grape berries. This study provides a theoretical foundation for elucidating the specific roles of PCD-related and *ATG* genes in seed abortion, offering candidate gene resources for the marker-assisted selection and breeding of seedless grapes.

## 2. Results

### 2.1. Morphological Changes During Fruit Development and Seed Abortion in the ‘Jingkejing’ Grape

**Figure 1:**
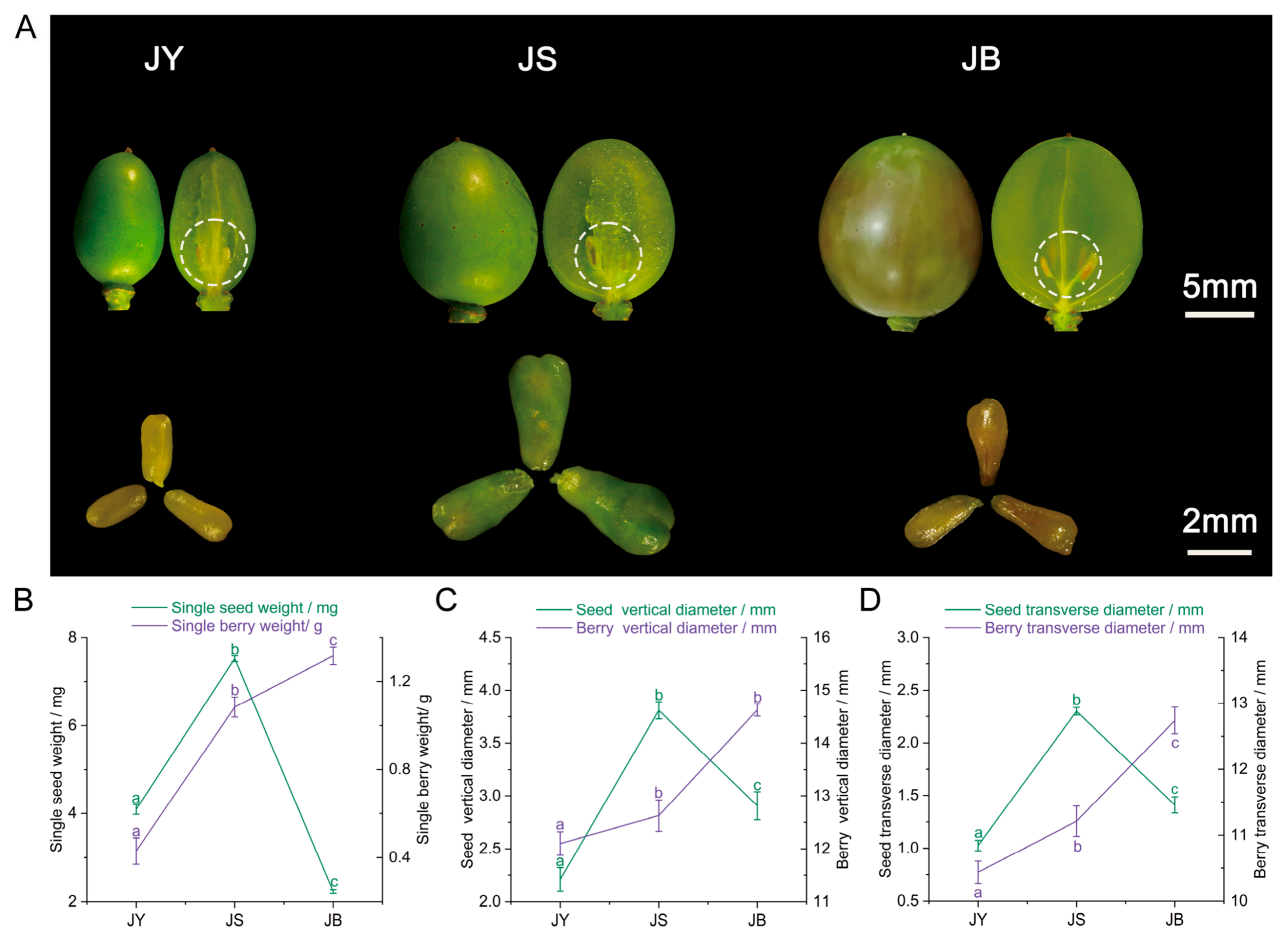
Morphological changes during berry development and seed abortion in ‘Jingkejing’: (**A**) Phenotypic characteristics of berries and seeds in the ‘Jingkejing’ grape at three critical developmental stages (white dashed circles indicate seed traces). (**B**) Changes in single berry weight and single seed weight. (**C**) Changes in the longitudinal diameters of berries and seeds. (**D**) Changes in the transverse diameters of berries and seeds. Data are presented as the mean ± standard deviation (SD). Different lowercase letters indicate statistically significant differences between developmental stages, determined by one-way ANOVA followed by Tukey’s test (*p* < 0.05). JY, JS, and JB correspond to the young fruit, seed-hardening, and abortion stages, respectively.

To investigate the mechanisms underlying seed abortion in pseudo-parthenocarpic grapes, morphological and physiological analyses were conducted on the cultivar ‘Jingkejing’ across three critical developmental stages: the young fruit (JY), seed-hardening (JS), and abortion (JB) stages. From the JY to the JB stages, single berry weight, along with longitudinal and transverse diameters, increased continuously (Figure 1B–D). Additionally, the berries began to undergo veraison (color transition) during the JB stage (Figure 1A). From the JY to the JS stages, single seed weight and dimensions (longitudinal and transverse diameters) increased gradually, peaking at the JS stage. However, by JB stage, the seeds exhibited pronounced shriveling and discoloration (Figure 1A). Concurrently, single seed weight dropped sharply, accompanied by significant reductions in both diameters (Figure 1B–D). These characteristic hallmarks of seed degradation and abortion reflect complex physiological shifts at the cellular level. Collectively, these findings suggest that grape seed abortion is likely associated with the critical molecular regulation of extensive PCD and active substance degradation processes.

### 2.2. Identification and Characterization of PCD-Related and ATG Gene Families in Grapevine

#### 2.2.1. Identification and Chromosomal Localization of PCD-Related and ATG Gene Families

**Figure 2:**
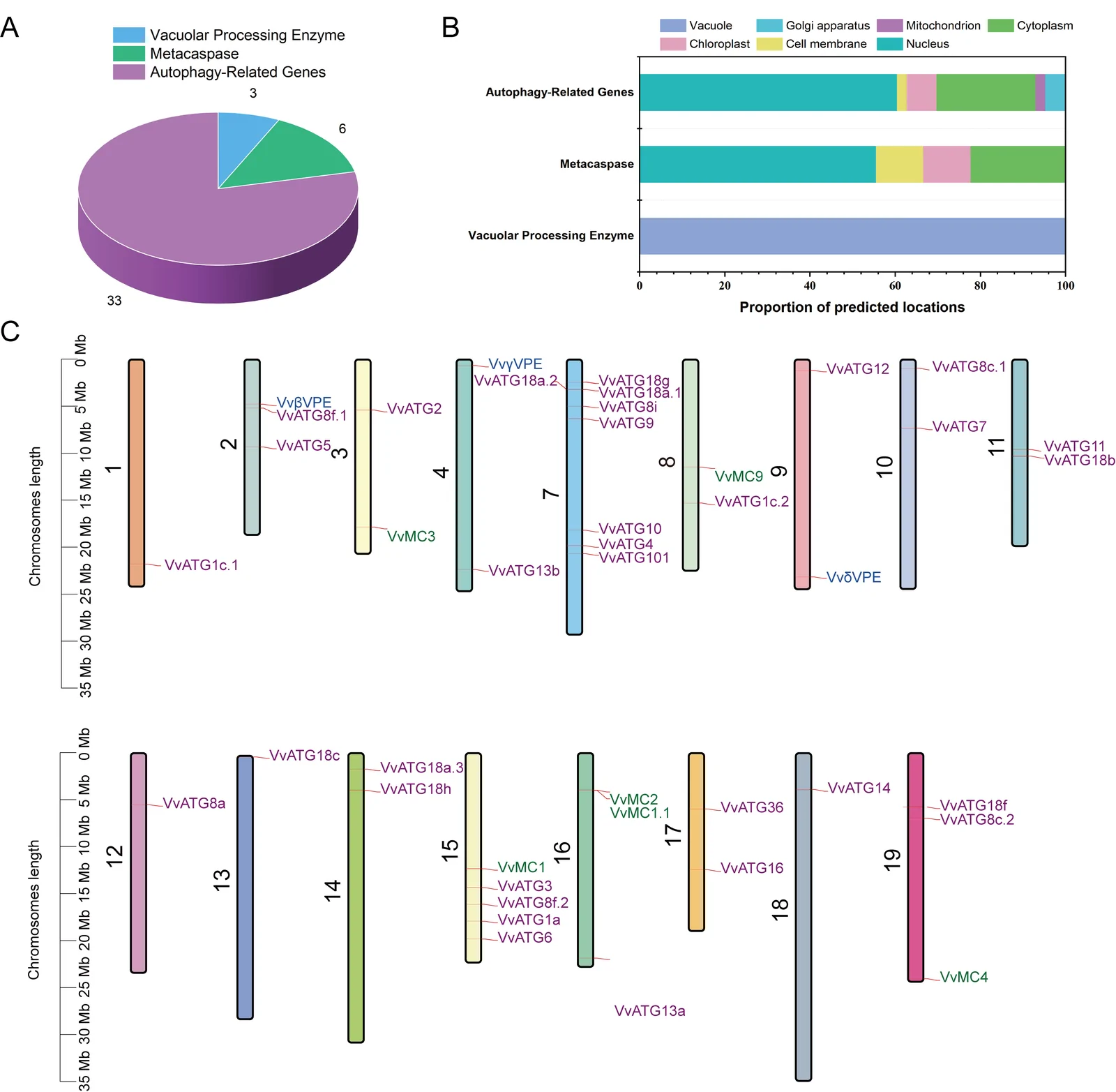
Identification and chromosomal localization of programmed cell death (PCD)-related and autophagy-related (ATG) gene families in grapevine: (**A**) Number of members within the PCD-related and *ATG* gene families in grapevine. (**B**) Subcellular localization of the PCD-related and ATG proteins. (**C**) Chromosomal localization of the PCD-related and *ATG* genes. Blue, green, and purple represent the *VPE*, *MC*, and *ATG* genes, respectively.

In this study, nine PCD-related genes (including three *VPE*s and six *MC*s) and 33 *ATG* genes were systematically identified in the grapevine genome (Figure 2A). Their basic characteristics, including chromosomal coordinates, protein lengths, molecular weights (MWs), isoelectric points (pIs), and predicted subcellular localizations (Table S1).

The VvVPE proteins exhibited similar MWs and pIs and were all predicted to localize to the vacuole (Figure 2B). The VvMC proteins exhibited MWs ranging from 34.88 to 46.25 kDa, with pIs from 5.05 to 8.75; all were predicted to localize to be nucleu-location, except for the cytoplasmic VvMC9 (Figure 2B). The VvATG proteins displayed substantial variation in length (94 to 2020 amino acids), MW (10.52 to 221.63 kDa), and pI (4.73 to 9.30), with predicted subcellular localizations spanning chloroplasts, mitochondria, the cytoplasm, the Golgi apparatus, and the nucleus (Table S1). This diversity likely reflects functional specialization within the ATG protein family (Figure 2B). Notably, protein lengths within the specific subfamily (e.g., the ATG8 subfamily: 119–129 aa) were highly conserved.

Chromosomal mapping revealed an uneven distribution of the 42 candidate genes across 17 of the 19 grapevine chromosomes (Figure 2C). Among the PCD-related genes, *VvMC1.1* and *VvMC2* mapped to the same chromosome, whereas the remaining genes were widely dispersed. The *ATG* genes were predominantly clustered on chromosomes 2, 7, 15, 16, and 19. Notably, several PCD-related genes and *ATG* genes co-mapped to the same chromosomal regions, suggesting potential functional or regulatory interactions.

#### 2.2.2. Gene Structure and Conserved Domain Architecture of PCD-Related and ATG Genes

**Figure 3:**
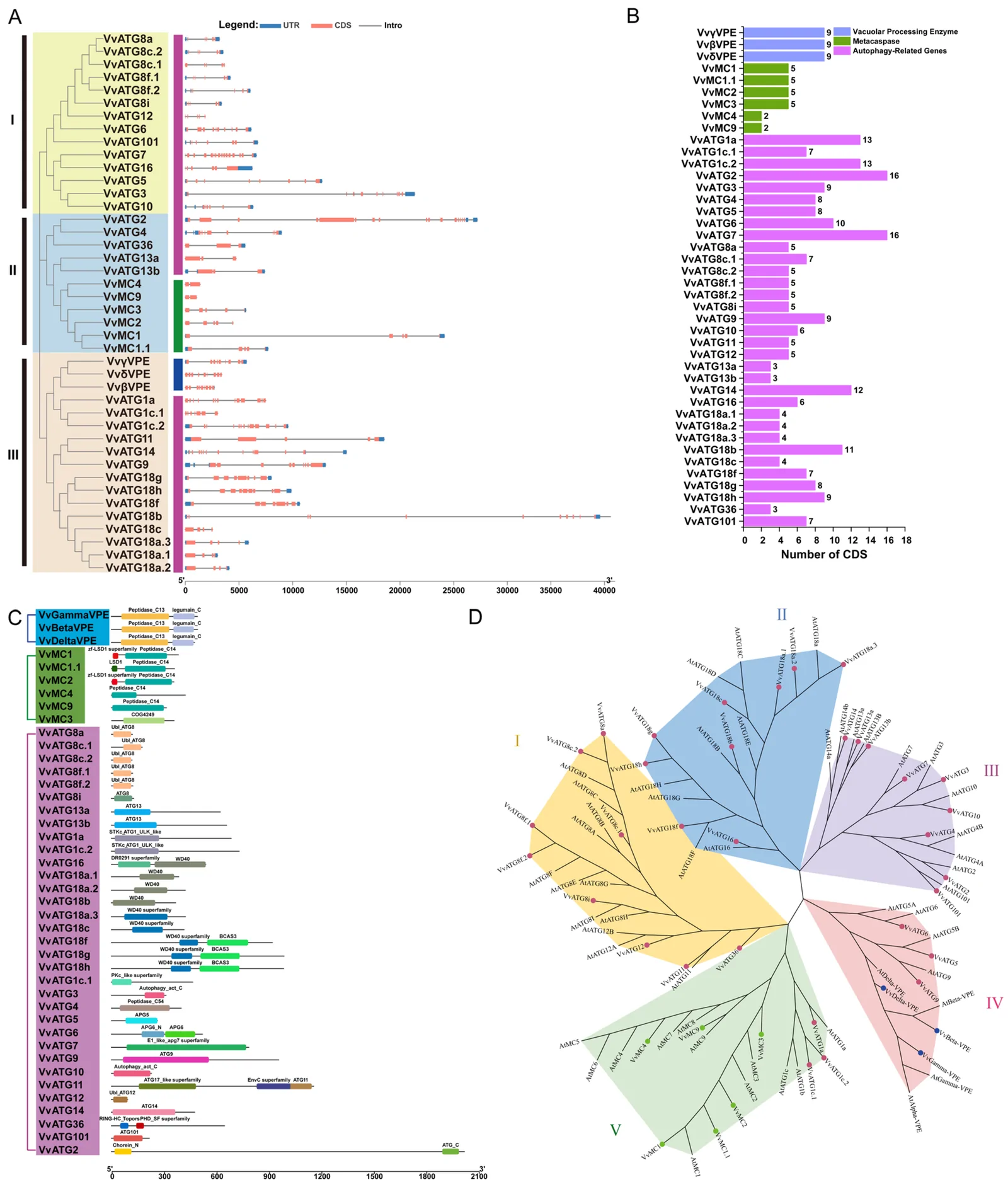
Bioinformatics analysis of PCD-related and *ATG* genes in grapevine: (**A**) Gene structures of the PCD-related and *ATG* genes in grapevine. The scale bar represents 5 kb. All genes are divided into Groups I, II, and III. (**B**) Number of coding sequences (CDSs) in the grapevine PCD-related and *ATG* genes. (**C**) Conserved domains of the grapevine PCD-related and *ATG* proteins. The scale bar represents 300 aa. Blue, green, and purple represent the vacuolar processing enzyme (VPE), metacaspase (MC), and ATG proteins, respectively. (**D**) Phylogenetic tree of the PCD-related and *ATG* gene families from grapevine and *Arabidopsis*. All proteins from the two species are clustered into five groups. Blue, green, and purple represent the VPE, MC, and ATG genes, respectively.

Gene structure analysis revealed significant variation in exon and intron numbers among the identified PCD-related and *ATG* genes in grapevine. Notably, genes clustered within the same phylogenetic clade exhibited highly similar structural features (Figure 3A). For instance, members of Group II contained fewer exons, whereas those in Groups I and III generally possessed more (Figure 3B). Thirty-three distinct conserved protein domains were identified across the 42 target proteins (Figure 3C). All VvVPE proteins contained both the Peptidase_C13 and legumain_C domains. Among the metacaspases, all members except VvMC3 possessed the typical Peptidase_C14 domain (Figure 3C). Within the ATG protein family, members of the same subfamily typically shared identical or similar domain compositions. For instance, all ATG13 subfamily members contained the conserved ATG13 domain, and all ATG8 subfamily members, except VvATG8i, possessed the Ubl_ATG8 domain. Notably, within the ATG18 subfamily, the protein lengths of VvATG18f, VvATG18g, and VvATG18h were approximately twice those of other members. Domain analysis further revealed an additional BCAS3 domain at their C-termini, suggesting that these proteins may perform specialized biological functions (Figure 3C).

#### 2.2.3. Phylogenetic Relationships of PCD-Related and ATG Gene Families

Phylogenetic analysis revealed that the 94 orthologous genes from grapevine and *Arabidopsis thaliana* clustered into five distinct groups (Figure 3D). Groups I and V were the largest: Group I comprised 21 *ATG* genes, while Group V included 15 *MC* and six *ATG* genes. In contrast, Group IV was the smallest, comprising only seven *VPE* and seven *ATG* genes. Overall, both PCD-related and *ATG* genes in grapevine exhibited highly conserved evolutionary relationships with their *Arabidopsis* counterparts. Moreover, the distribution patterns of the *VPE* and *MC* families within the phylogenetic tree mirrored those of the *ATG* family, suggesting a parallel evolutionary trajectory.

#### 2.2.4. Genomic Synteny and Duplication Events of PCD-Related and ATG Gene Families

**Figure 4:**
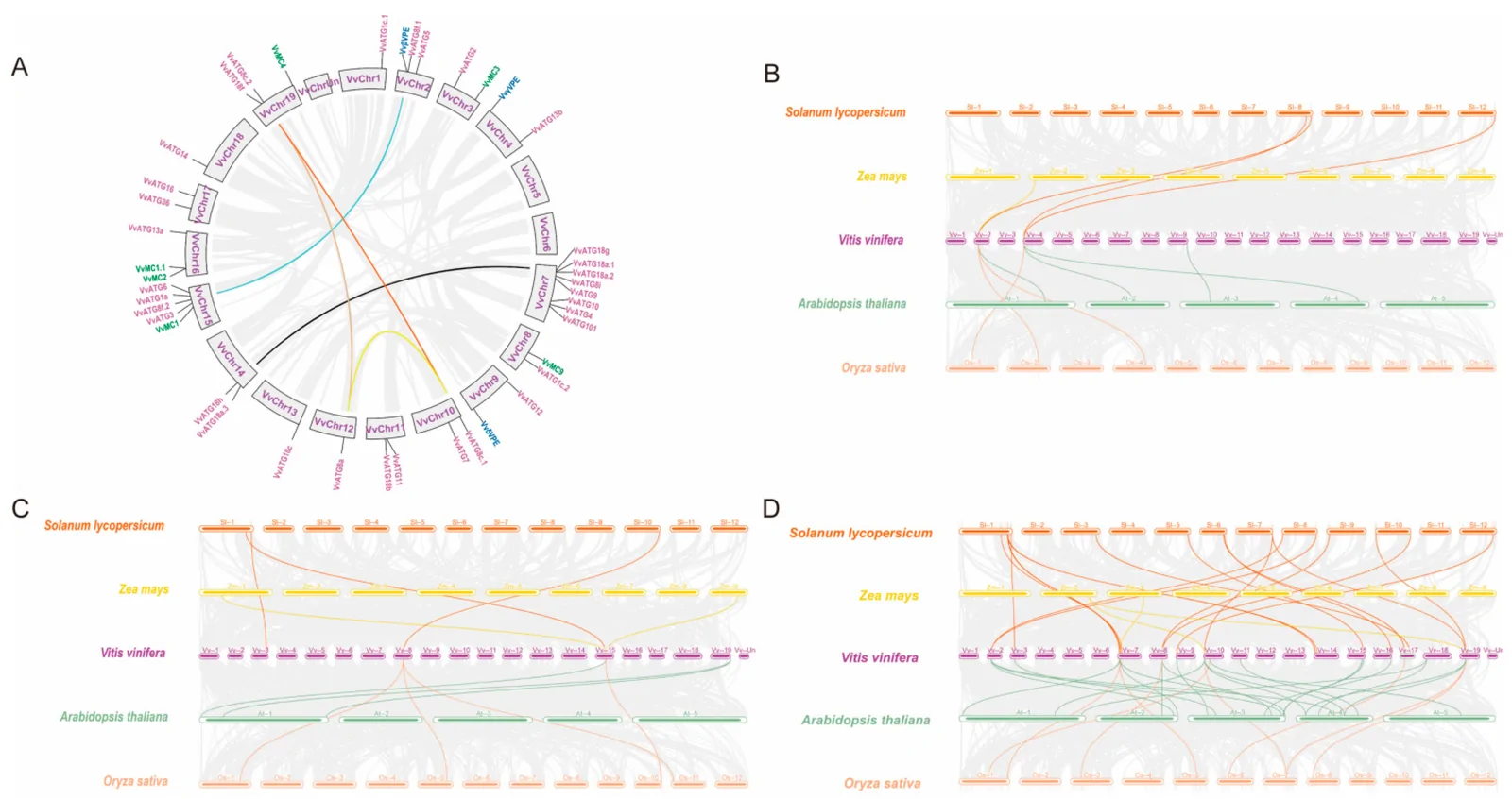
Collinearity analysis of the PCD-related and *ATG* gene families in grapevine: (**A**) Intraspecific collinearity analysis of PCD-related and *ATG* genes within the grapevine genome. Background gray lines indicate the collinear blocks within the entire grapevine genome, whereas colored lines highlight the collinear pairs among the candidate genes. (**B**–**D**) Interspecific collinearity analysis between grapevine and four other plant species (*Solanum lycopersicum*, *Zea mays*, *Arabidopsis thaliana*, and *Oryza sativa*). (**B**) *VPE* gene family. (**C**) *MC* gene family. (**D**) *ATG* gene family. Gray lines represent the collinear blocks between the respective plant genomes, whereas colored lines indicate the identified interspecific orthologous gene pairs.

To investigate the evolutionary patterns of PCD-related and *ATG* genes in grapevine, we performed intraspecific collinearity analysis on the 42 candidate genes. Five collinear gene pairs were identified, all within the ATG family, indicating that gene duplication contributed to its expansion (Figure 4A). *Ka*/*Ks* analysis further revealed that all five pairs exhibited ratios < 1, suggesting they have undergone purifying selection (Table 1). This stringent evolutionary constraint implies that the core structural and functional integrity of these proteins is highly conserved and indispensable. During seed abortion, maintaining this conserved autophagic and PCD machinery ensures the reliable execution of extensive cellular dismantling and nutrient remobilization.

We next examined interspecific collinearity between grapevine and four other species—*Arabidopsis thaliana*, *Solanum lycopersicum*, *Oryza sativa*, and *Zea mays*. Grapevine shared four (*VPE*) and 22 (*ATG*) collinear gene pairs with *Arabidopsis*, which were significantly higher than those observed with the other three species (Figure 4B,D). For *MC* genes, grapevine exhibited four, three, three, and two collinear pairs with *Solanum lycopersicum*, *Arabidopsis thaliana*, *Oryza sativa*, and *Zea mays*, respectively (Figure 4C). Notably, *VvMC1.1* and *VvMC2* exhibited no collinearity, suggesting they may be grapevine-specific or have undergone structural variations (Figure 4C). Collectively, these results indicate that grapevine *VPE* and *ATG* genes share a closer evolutionary relationship with *Arabidopsis thaliana*, whereas the *MC* gene family displays a distinct pattern.

### 2.3. Protein–Protein Interaction Networks of PCD-Related and ATG Proteins

The PPI networks of the PCD-related (VPE and MC) and ATG proteins in grapevine were predicted using the STRING database. The results revealed extensive interactions among the 28 ATG proteins, whereas no direct intrafamilial interactions were observed within the VPE or MC families. Instead, these proteins were predicted to interact with diverse array of functional proteins.

**Figure 5:**
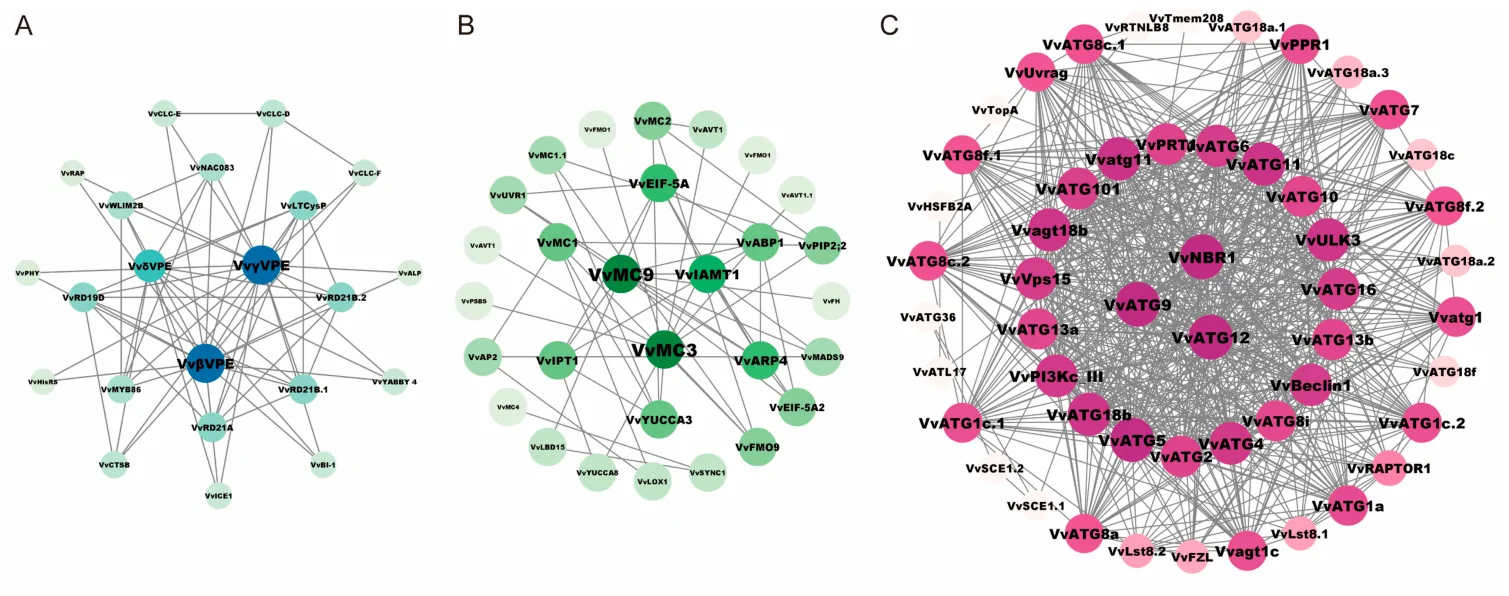
Protein–protein interaction networks of PCD-related and ATG protein families in grapevine: (**A**) Interaction network of VvVPE proteins. (**B**) Interaction network of VvMC proteins. (**C**) Interaction network of VvATG proteins.The blue, green, and purple dots indicate VPE, MC, and ATG proteins, respectively. Darker colors and larger dots represent a higher degree of interaction.

VPE proteins exhibited direct interactions with multiple target proteins, including a bHLH transcription factor (VvICE1), an LIM protein (VvWLIM2B), a membrane protein (BI-1), an NAC transcription factor (VvNAC083), a YABBY transcription factor (VvYAB1), and several cysteine proteases (VvCT-SB, VvRD21A, VvRD21B.1, VvRD21B.2, and VvOs04g0650000) (Figure 5A). These findings suggest that VPE proteins are broadly involved in abiotic stress responses (e.g., drought and low temperature), hormone signal transduction, and plant morphogenesis, thereby exerting pleiotropic regulatory effects on diverse physiological processes in grapevine. Within the MC family, four members were predicted to interact directly with three specific proteins: an aquaporin (VvPIP2.2) and two flavin-containing monooxygenases (VvYUCCA3 and VvFMOGS-OX9). This suggests that the MC proteins play crucial roles in water uptake, H_2_O_2_ signaling, and plant development, particularly through auxin biosynthesis (Figure 5B).

In the ATG family, VvATG9, VvNBR1, and VvATG12 interacted with the majority of network proteins, acting as core hubs. As key components of the target of rapamycin (TOR) complex, the VvRAPTOR1 and VvLST8 mediate extensive crosstalk across hormonal, metabolic, and signaling pathways. Additionally, PRT1 is essential for maintaining physiological stability through the regulation of protein homeostasis. Furthermore, the integration of the heat stress-responsive VvHSFB2a and the root-expressed VvPI3KC_III underscores the multifaceted role of autophagy in stress adaptation and developmental regulation (Figure 5C).

### 2.4. Spatiotemporal Expression Profiles of PCD-Related and ATG Gene Families

**Figure 6:**
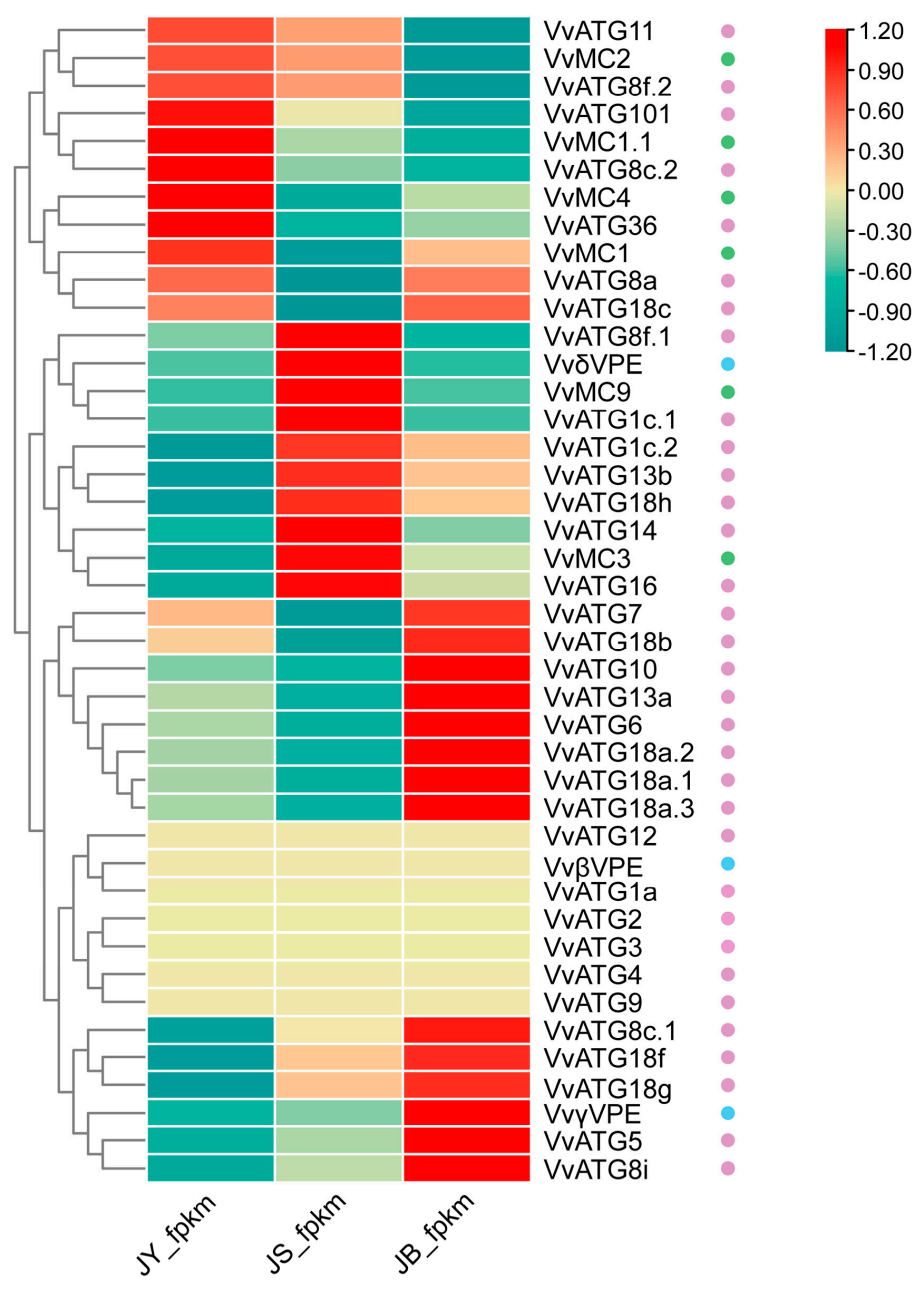
Expression heatmap of PCD-related and *ATG* genes across various developmental stages in grapevine. The blue, green, and purple dots indicate *VPE*, *MC*, and *ATG* genes, respectively. The abbreviations JY, JS, and JB represent the young fruit, seed-hardening, and seed abortion stages, respectively.

To profile the global transcriptional dynamics during seed abortion, differentially expressed genes (DEGs) among the JY, JS, and JB stages were identified using standard false discovery rate (FDR) correction (Figure S1). We then evaluated the DEG status of the PCD-related and ATG gene families within these comparison groups (Table S4). To group genes with similar spatiotemporal trajectories, we hierarchically clustered their expression profiles and visualized the results as a dendrogram (Figure 6). Most PCD-related genes maintained high expression levels throughout seed development. Notably, *VvγVPE* exhibited a gradually increase in expression, whereas *VvδVPE* and *VvMC9* peaked at the seed-hardening stage compared to the young fruit and abortion stages, suggesting their involvement in seed development up to hardening and the subsequent initiation of abortion. Within the *ATG* family, expression patterns varied considerably; several members showed minimal variation across stages, whereas others exhibited dynamic or highly stage-specific expression. Specifically, *VvATG1c.2*, *VvATG8a*, *VvATG11*, *VvATG13a*, *VvATG13b*, and multiple *VvATG18* members (*VvATG18a.1*, *VvATG18a.2*, *VvATG18a.3*, *VvATG18f*, *VvATG18g*, and *VvATG18h*) were highly expressed, suggesting crucial roles during seed abortion. Furthermore, *VvATG5*, *VvATG8c.1*, *VvATG8i*, *VvATG18f*, and *VvATG18g* were continuously upregulated throughout development, implying a positive regulatory role in seed abortion, whereas *VvATG8c.2*, *VvATG8f.2*, *VvATG11*, and *VvATG101* were continuously downregulated, suggesting negative regulation.

Additionally, *VvATG*6, *VvATG7*, *VvATG18a.1*, *VvATG18a.2*, and *VvATG18a.3* initially decreased but were markedly upregulated from the hardening to the abortion stages, likely reflecting a heightened demand for intracellular degradation and recycling during late abortion. Conversely, *VvATG1c.1*, *VvATG1c.2*, *VvATG8f.1*, *VvATG13b*, *VvATG14*, *VvATG16*, and *VvATG18h* displayed an initial increase followed by a decrease, with *VvATG8f.1* showing pronounced upregulation at the hardening stage, indicating that these genes function primarily during the early response to abortion signals or at the critical hardening stage.

### 2.5. Potential Functional Analysis of PCD-Related and ATG Genes During Grape Seed Abortion

These transcriptomic profiles indicate that the PCD-related and *ATG* genes exhibit highly specific spatiotemporal expression patterns during grapevine seed abortion. To further elucidate the upstream molecular mechanisms driving the dynamic transcriptional reprogramming of these candidate genes, we analyzed their promoter regions to identify key *cis*-regulatory elements and potential transcription factor binding sites.

#### 2.5.1. Promoter Architecture and Cis-Regulatory Elements of PCD-Related and ATG Gene Families

**Figure 7:**
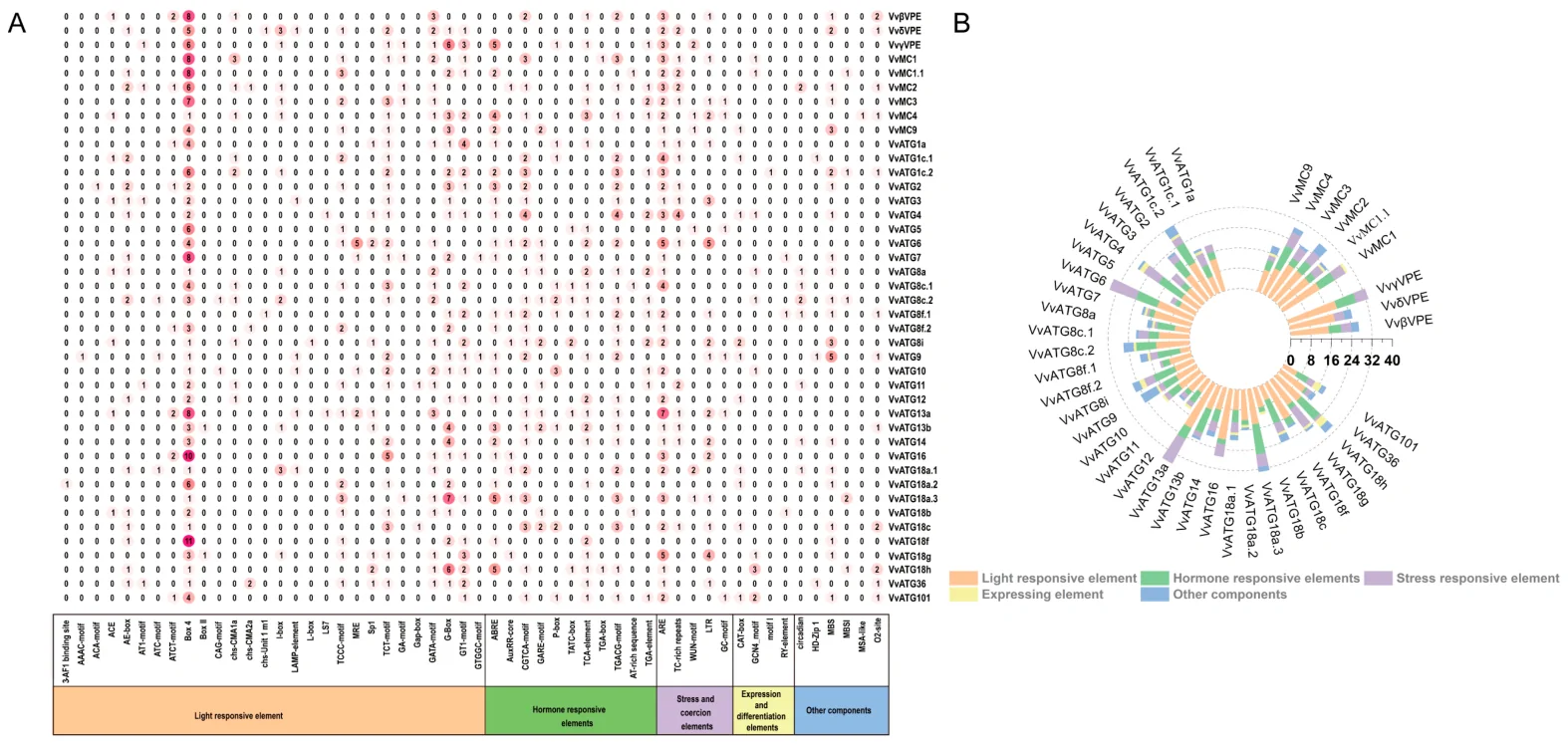
Prediction of cis-acting elements in the PCD-related and *ATG* gene families in grapevine: (**A**) The number of different cis-acting elements in the *VPE*, *MC*, and *ATG* genes. The color intensity (from white to red) in the matrix reflects the abundance of each cis-acting element, with a darker red indicating a higher number.(**B**) The number of cis-acting elements across five functional categories in the *VPE*, *MC*, and *ATG* gene families.

Using the PlantCARE database, we predicted *cis*-acting elements in the promoter regions (2 kb upstream of the start codon) of the 42 target genes. These elements were classified into five categories: light-responsive elements (e.g., TCT-motif, AE-box, GT1-motif, G-Box, Box 4, GATA-motif, and MRE), hormone-responsive elements (gibberellin: GARE-motif, P-box, TATC-box; methyl jasmonate: TGACG-motif, CGTCA-motif; abscisic acid: ABRE; auxin: AuxRR-core; salicylic acid: TCA-element), stress-responsive elements (TC-rich repeats, LTR, GC-motif, MBS, WUN-motif, and ARE), tissue-specific responsive elements (GCN4_motif, RY-element, CAT-box, and motif I), and other elements (e.g., circadian control) (Figure 7A,B).

Light-responsive elements were the most abundant, followed by hormone-responsive elements. Among the 42 genes, all but *VvδVPE* contained hormone-responsive elements. Notably, the promoters of *VvATG6* and *VvATG8f.1* harbored all five types of hormone-responsive elements, suggesting they play central roles in hormone signaling in grapevine (Figure 7A). All PCD-related gene promoters contained stress-responsive elements. Within the *ATG* family, stress-responsive elements were detected in all members except *VvATG8c.2*, *VvATG18a.2*, *VvATG18b*, and *VvATG18f*, with the ARE motif being the most prevalent (Figure 7A). Additionally, tissue-specific regulatory elements, including the GCN4_motif (endosperm development), RY-element (seed development), CAT-box (meristem development), and motif I (root development), were identified in the promoters of 18 genes (Figure 7A).

#### 2.5.2. Screening and Prediction of Transcription Factors Targeting PCD-Related and ATG Genes

**Figure 8:**
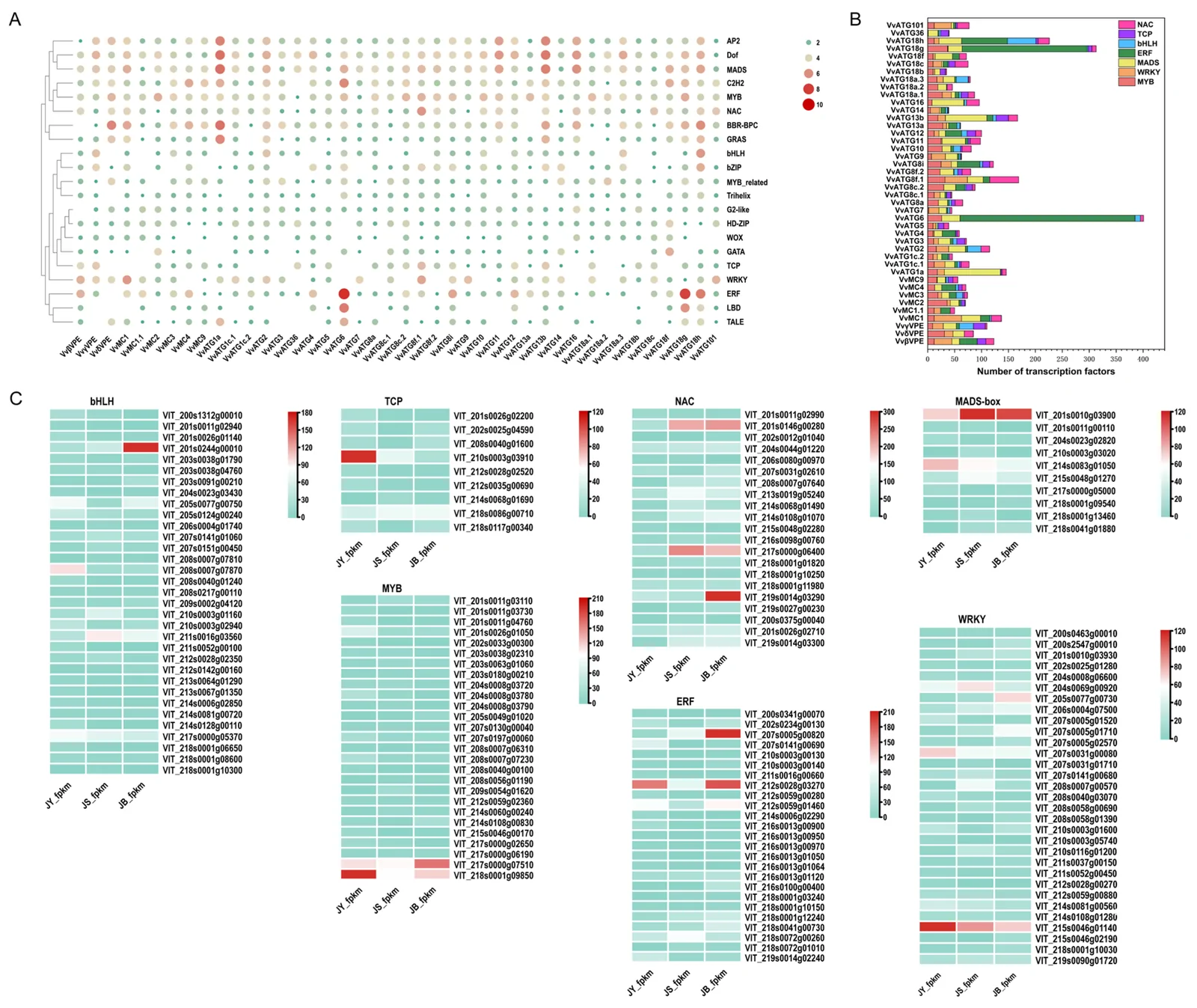
Identification of TFs regulating PCD-related and *ATG* genes in grapevine: (**A**) Transcription factor families identified in the promoter regions of PCD-related and *ATG* genes. (**B**) Classification and number of members within the transcription factor families involved in the regulation of PCD-related and *ATG* genes. (**C**) Expression heatmap of candidate transcription factor genes across various developmental stages. Lowly expressed genes were filtered out based on a threshold of mean FPKM < 5.

To identify potential upstream regulators of PCD-related and *ATG* genes, we predicted TF binding sites within their promoter regions (2 kb upstream of the ATG start codon) using the PlantRegMap database, identifying 21 TF families (Figure 8A). These families were broadly distributed across the promoters of PCD-related and *ATG* genes, with binding sites for the MYB, WRKY, NAC, and ERF families showing significant enrichment, suggesting that these TFs may play core roles in coordinating the transcriptional regulation of PCD and autophagy. From these 21 families, we selected seven with highly represented binding sites for further analysis. Comparative analysis revealed that the ERF family putatively regulates the largest number of PCD-related and *ATG* genes, whereas the MYB and MADS families were predicted to regulate all target genes (Figure 8B). Integrating these predictions with transcriptome data revealed that these seven TF families exhibited diverse expression patterns during seed abortion. Among the highly expressed TFs, VIT_215s0046g01140 (*VvWRKY46*), VIT_214s0083g01050 (*VvSEP1*), and VIT_210s0003g03910 (*VvTCP2*) showed progressive downregulation throughout seed abortion, whereas VIT_207s0005g00820 (*VvERF71*), VIT_201s0146g00280 (*VvNAC83*), and VIT_201s0244g00010 (*VvbHLH137*) were continuously upregulated. Additionally, several TFs displayed phasic fluctuations: VIT_217s0000g06400 (*VvNAC100*) and VIT_201s0010g03900 (*VvFBP2*) increased initially before declining, whereas VIT_218s0001g09850 (*VvMYB44*), VIT_217s0000g07510 (*VvMYB1R1*), VIT_212s0028g03270 (*VvERF4*), and VIT_219s0014g03290 (*VvNAC72*) initially decreased but were subsequently upregulated (Figure 8C).

#### 2.5.3. Potential Hormonal Regulation of PCD-Related and ATG Promoters During Grape Seed Abortion

**Figure 9:**
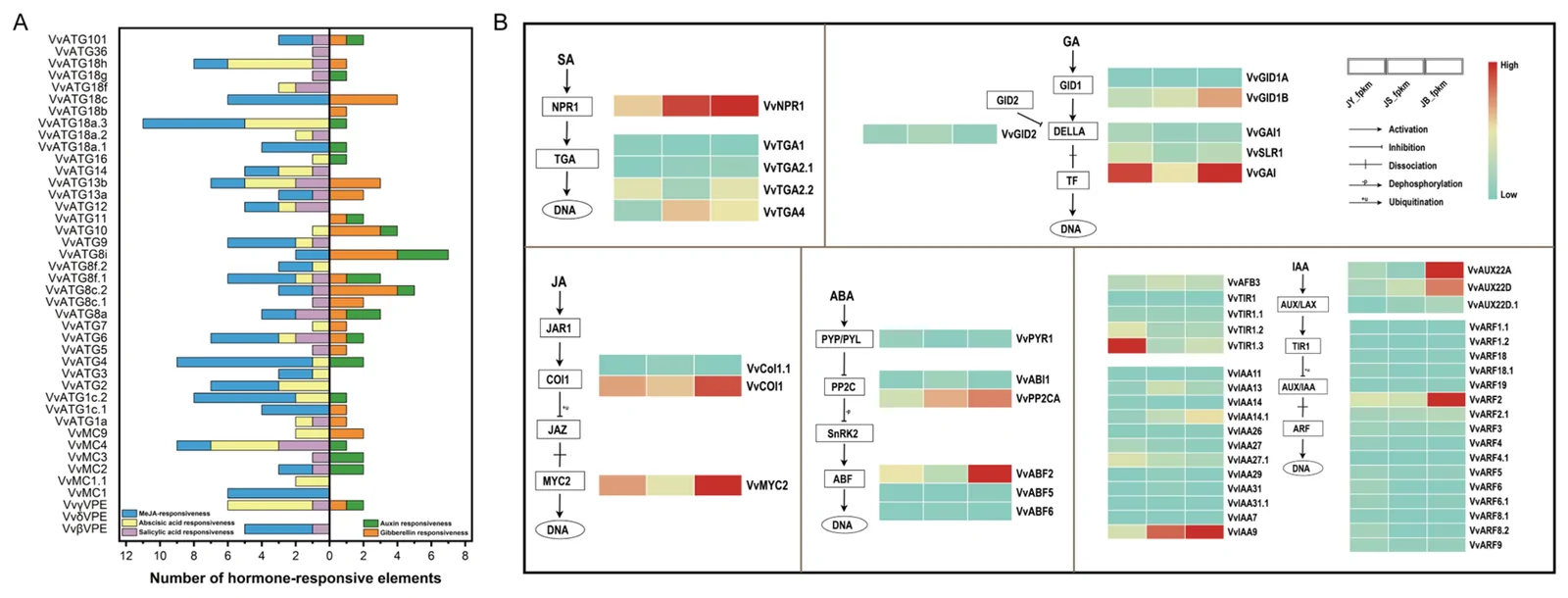
Potential hormone-responsive pathways of PCD-related and *ATG* genes in grapevine: (**A**) Classification and number of hormone-responsive elements identified in the promoter regions of PCD-related and *ATG* genes. (**B**) Expression heatmap of key genes involved in the GA, IAA, SA, JA, and ABA signaling pathways across various developmental stages.

Hormone signaling plays a pivotal role in seed abortion. Based on the hormone-responsive elements identified in the promoters of PCD-related and *ATG* genes, we predicted potential hormone regulatory networks (Figure 9A). The promoters of *VvATG4*, *VvATG8i*, and *VvATG18a.3* were highly enriched with such elements, indicating pronounced hormone sensitivity, whereas *VvδVPE*, *VvMC1.1*, and *VvATG18b* were largely devoid of them. Jasmonic acid (JA)-responsive elements were the most widespread (present in 25 genes), suggesting that JA signaling serves as a primary hormonal cue. Abscisic acid (ABA) and salicylic acid (SA) responsive elements were each present in 23 genes, although the total number of SA elements was comparatively lower. Indole-3-acetic acid (IAA) and gibberellin (GA) elements were identified 18 and 20 genes, respectively, with IAA elements being the least abundant. The *VvATG8* subfamily generally harbored highly diverse hormone regulatory elements.

We next analyzed the expression patterns of key pathway genes during seed abortion (Figure 9B). In the SA pathway, the expression of *VvNPR1* gradually increased, paralleling abortion progression. In the JA pathway, the expression levels of *VvCOI1* and the downstream gene *VvMYC2* both rose from the JS to JB stages, indicating sustained JA signaling amplification during late abortion. In the ABA pathway, the expression of *VvABF2* peaked at the JB stage, marking a critical period for ABA responses. In the GA pathway, the expression of *VvGID1B* gradually increased, promoting DELLA degradation, whereas *VvGAI* initially decreased before increasing, reflecting dynamic GA regulation with strong suppression at the JB stage. In the IAA pathway, VvTIR1.3 expression peaked at the young fruit stage (JY), whereas the negative regulators *VvIAA9*, *VvAUX22A*, and *VvAUX22D* all peaked at the JB stage, suppressing auxin signaling during late abortion. Notably, *VvARF2* expression mirrored that of *VvIAA9*, suggesting synergistic negative feedback regulation.

#### 2.5.4. A PCD/ATG-Mediated Regulatory Network Orchestrated by Transcription Factors and Hormones During Seed Abortion in Pseudo-Parthenocarpy Grape

**Figure 10:**
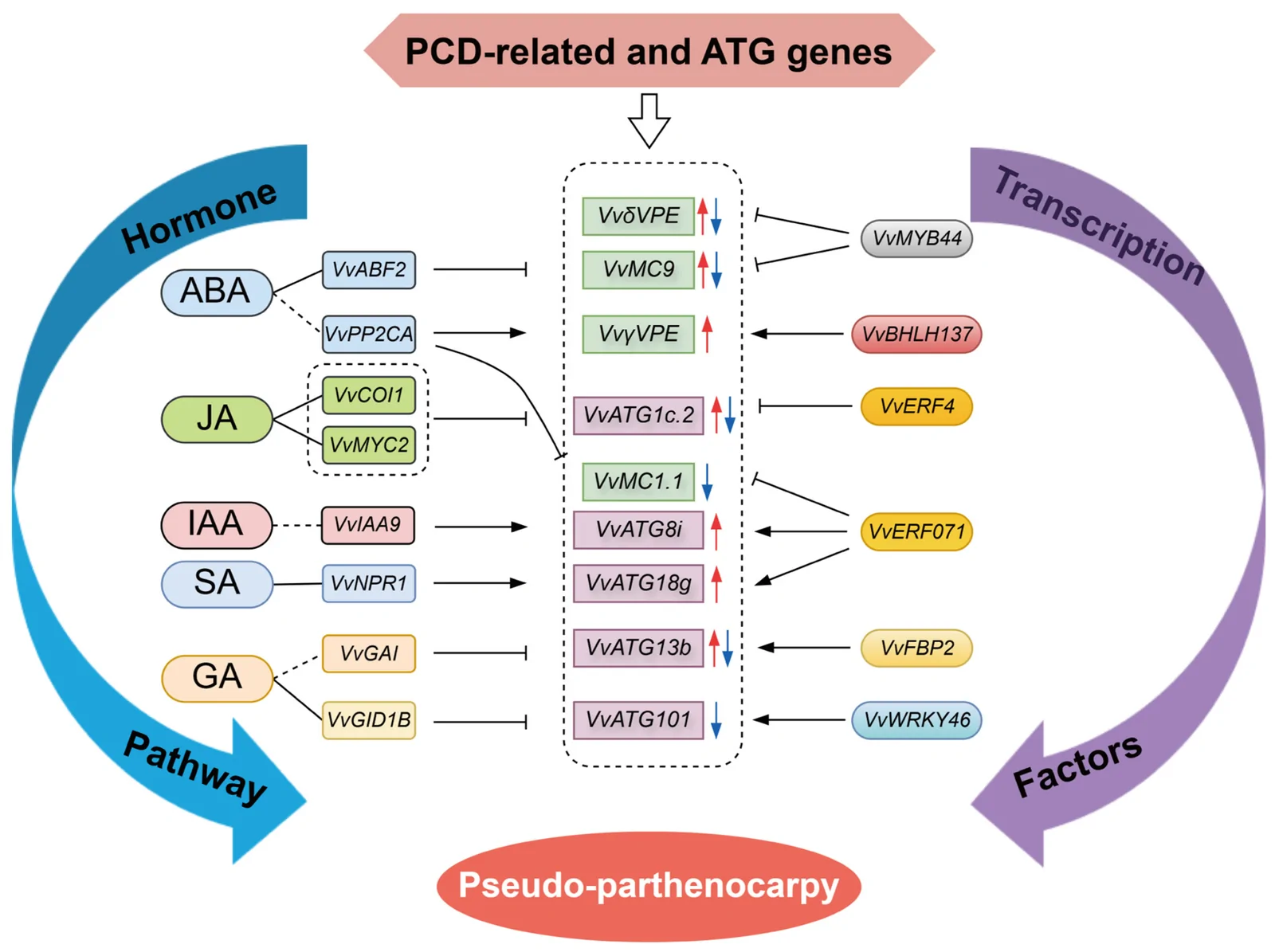
Transcriptional and hormonal regulatory network mediating PCD and autophagy during seed abortion in pseudo-parthenocarpy grape. The central panel highlights core PCD-related and *ATG* genes, with red upward and blue downward arrows indicating their dynamic upregulation and downregulation, respectively, during seed abortion. The left panel depicts key regulators from various hormone signaling pathways, including abscisic acid (ABA), jasmonic acid (JA), indole-3-acetic acid (IAA), salicylic acid (SA), and gibberellic acid (GA), while the right panel displays the associated transcription factors. Solid lines with arrowheads indicate positive regulation, whereas solid blunt-ended lines represent negative regulation between the regulators and their target genes. Between hormones and their downstream signaling components, solid lines denote positive correlations, while dashed lines indicate negative correlations.

To further elucidate the potential regulatory mechanisms underlying pseudo-parthenocarpy, a co-expression network integrating hormone signaling regulators, transcription factors, and core PCD-related/ATG genes was constructed (Figure 10). Regarding hormone signaling pathways, the positive regulator of SA signaling, VvNPR1, was positively correlated with the upregulated gene VvATG18g. This suggests that SA signaling may promote autophagic degradation during seed abortion. Similarly, VvIAA9, a negative regulator of the IAA signaling pathway, exhibited a positive correlation with the upregulated gene VvATG8i, implying that the attenuation of auxin signaling may contribute to the induction of autophagy. In contrast, key components of the JA signaling pathway (VvCOI1 and VvMYC2), the ABA pathway regulator VvABF2, and the negative regulator of GA signaling VvGAI were predominantly negatively correlated with downregulated genes such as VvATG1c.2, VvδVPE, and VvATG13b. These results indicate that distinct hormone signaling pathways coordinately regulate the expression of PCD-related and ATG genes, thereby driving cellular fate transitions and the progression of seed abortion. Furthermore, transcription factors appear to bridge these signaling pathways within the regulatory network. Notably, VvERF71 was positively correlated with the ATG genes VvATG8i and VvATG18g but negatively correlated with VvMC1.1, suggesting a potential role in balancing autophagy and PCD during seed abortion. Additionally, synergistic positive correlations were observed between VvbHLH137 and VvγVPE, VvFBP2 and VvATG13b, as well as VvWRKY46 and VvATG101. Conversely, VvMYB44 was negatively correlated with VvδVPE and VvMC9, while VvERF4 showed a negative correlation with VvATG1c.2. These findings suggest that diverse transcription factor families may differentially regulate PCD-related and ATG genes, collaboratively mediating the protein degradation and cell death processes intrinsic to seed abortion.

### 2.6. Functional Verification of Transcription Factor Regulation on PCD-Related and ATG Genes

**Figure 11:**
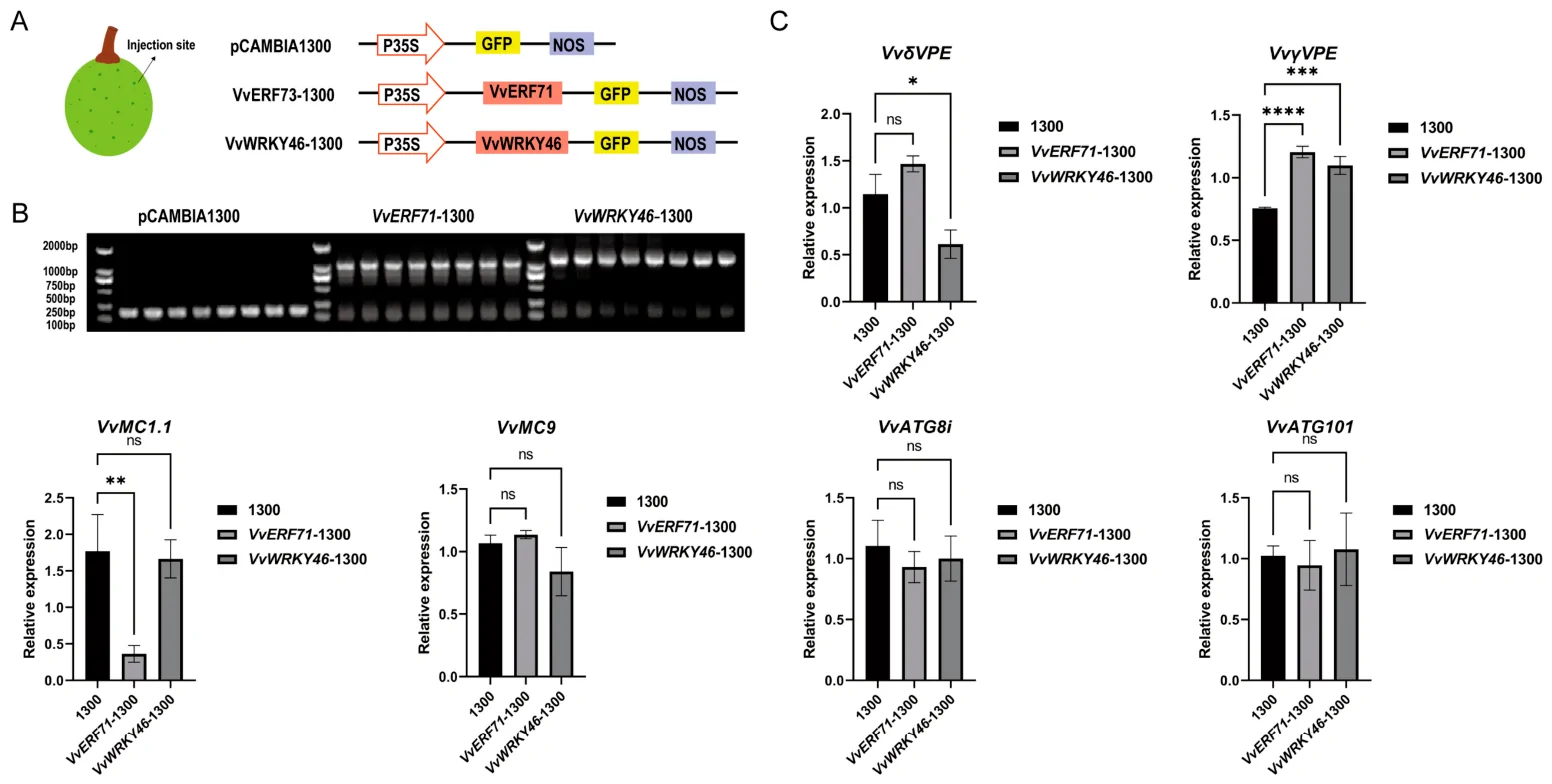
Effects of *VvWRKY46* and *VvERF71* overexpression on the expression of PCD-related and *ATG* genes in transiently transformed grape fruits: (**A**) Schematic diagram of *Agrobacterium*-mediated transient injection into grape berries and the structure of the overexpression vectors. (**B**) PCR-based positive identification of the transiently transformed grape fruit tissues. (**C**) qRT-PCR analysis of the expression changes in PCD-related and *ATG* target genes following the overexpression of *VvWRKY46* and *VvERF71*. The empty vector pCAMBIA1300 (labeled as 1300) served as the negative control to establish baseline expression. Data are presented as the mean ± standard deviation (SD). ns, not significant. * *p* < 0.05, ** *p* < 0.01, *** *p* < 0.001, **** *p* < 0.0001.

Based on promoter *cis*-acting elements and transcription factor binding site prediction, we identified a significant enrichment of the WRKY and ERF transcription factor families in the regulatory networks of the target genes. To experimentally validate these predictions, we selected two transcription factors exhibiting opposite expression trends during seed abortion—the progressively downregulated *VvWRKY46* and the progressively upregulated *VvERF71*—along with six PCD-related and ATG genes that showed high expression during critical developmental stages and contained WRKY or ERF binding sites in their promoters as potential downstream targets. Using an Agrobacterium-mediated transient expression system, we individually overexpressed *VvWRKY46* and *VvERF71* in grape fruits (Figure 11A). PCR verification confirmed the successful introduction of the empty vector control (pCAMBIA1300) and both overexpression constructs (*VvERF71*-1300 and *VvWRKY46*-1300) into the grape fruits (Figure 11B). The empty vector (pCAMBIA1300) served as the negative control. Subsequently, qRT-PCR was employed to assess their effects on the six candidate target genes relative to the basal expression levels of this control (Figure 11C).

The overexpression of *VvERF71* significantly upregulated the PCD-related genes *VvVPE* and *VvγVPE* (with the latter showing a highly significant increase) while suppressing *VvMC1.1*, suggesting that *VvERF71* may regulate PCD in grape embryos by activating the VPE pathway and repressing certain metacaspase-related genes. Conversely, *VvWRKY46* overexpression significantly inhibited *VvVPE* but resulted in a highly significant upregulation of *VvγVPE*, whereas VvMC9 showed a non-significant downward trend, indicating that VvWRKY46 exerts differential regulatory effects on distinct PCD-related genes. Notably, neither VvERF71 nor VvWRKY46 significantly altered the expression of the autophagy-related genes *VvATG8i* and *VvATG101*, implying that their regulatory impact on the autophagy pathway may be relatively weak or unrelated to the direct modulation of *ATG* gene expression. Collectively, these results further substantiate the important roles of the WRKY and ERF transcription factors within the regulatory network governing grape seed abortion, as well as their specific regulatory effects on downstream targets.

## 3. Discussion

During seed development, the spatiotemporal coordination of PCD and autophagy is essential. Dysregulation of these processes induces aberrant cell death and seed abortion. In grapevine, we identified three VPE members (*VvβVPE*, *VvγVPE*, and *VvδVPE*) that function as potential vacuole-mediated PCD executioners [6]. Consistent with previous reports [2], the seed-specific *VvβVPE* was downregulated during seed abortion, likely disrupting the nutrient supply. Conversely, the high constitutive expression of *VvγVPE* and the strong induction of *VvδVPE* at the seed-hardening stage indicate an abnormal activation of PCD signals promoting embryonic tissue degeneration [8,33,34]. Similarly, MCs serve as crucial PCD executioners during tissue development. Among the six identified grapevine *MC* members, *VvMC9* expression peaked sharply at the seed-hardening stage. Given that misregulated MC expression causes embryo arrest in model plants [7,15], this sudden upregulation suggests that *VvMC9* functions as a primary downstream executioner of the PCD cascade in seedless grapes. Autophagy also plays a dual role in plant reproduction [16,35]. We identified 33 grapevine *ATG* members, among which key hub genes (e.g., *ATG1*, *ATG8*) exhibit multi-copy expansion, likely enhancing their regulatory capacity [36,37]. Notably, ATG genes containing seed-specific regulatory elements (*VvATG7*, *VvATG8c.2*, *VvATG101*) exhibited significant dynamic expression changes during seed abortion.

Our protein-level analyses, integrating STRING PPI predictions with grapevine PCD and *ATG* gene data, demonstrate that these pathways do not operate in isolation but rather integrate into complex signaling networks involving phytohormones, vesicle trafficking, and early embryo development. Rather than interacting with one other, VPEs connect with distinct signaling proteins, notably the bHLH transcription factor ICE1 and the conserved PCD inhibitor BI-1 [38]. Because ICE1 is crucial for early embryo patterning [39] and the absence of BI-1 triggers vacuole-mediated cell death [6,40,41], we suggest that VPEs may promote seed abortion by interfering with these key developmental and survival regulators. The MC interaction network is predominantly enriched in hormone metabolism pathways. MCs are predicted to interact with YUCCA family members (e.g., VvYUCCA3), which are rate-limiting enzymes in auxin biosynthesis [42,43]. This physical interaction suggests that MCs might disrupt auxin homeostasis, a critical factor for normal seed development [44,45]. Additionally, MCs interact with the highly conserved translation elongation factor EIF-5A. Given that the loss of EIF-5A disrupts gametophyte and embryonic development [46], MC-mediated PCD might also involve the proteolytic cleavage and inhibition of fundamental translational machinery required for embryogenesis. Conversely, the *ATG* family forms a dense internal network (e.g., VvATG9, VvNBR1, and VvATG12 hubs) closely linked to VvRAPTOR1 and VvLST8, which are core components of the target of rapamycin (TOR) complex. As a central energy sensor, the TOR complex acts as an energy sensor that typically antagonizes autophagy [37,47]. Furthermore, the network includes VPS15, an essential kinase for autophagosome formation and vacuolar regulation [37,48]. We propose that during seed abortion, nutrient stress-induced inhibition of the TOR complex may activate VPS15-dependent vacuologenesis and autophagy [37,49]. This process likely synergizes with VPE-mediated tonoplast rupture to ultimately drive the degradation of embryonic tissues [6,33].

This protein-level crosstalk is further corroborated by transcriptional regulation. Our promoter and transcriptomic analyses suggest a multi-hormonal regulatory network governing PCD-related and *ATG* genes. The enrichment of hormone-responsive motifs (e.g., SA, GA, ABA, JA, and IAA) in their promoters aligns with the synchronized expression of key hormonal signaling hubs (*VvNPR1*, *VvMYC2*, and *VvABF2*). These findings imply that fluctuations in endogenous hormones likely act as upstream triggers for autophagy and PCD cascades during seed abortion [37,50]. This observation aligns with previous evidence that phytohormones such as ABA, GA, and SA can directly induce *VPE* expression [51,52], and that *ATG* genes dynamically respond to JA, SA, and ABA during stress [53,54]. In our study, the upregulation of genes involved in stress-related hormone pathway (e.g., JA, SA, and ABA) coincided with the transcriptional activation of *VPE*s, *MC*s, and *ATG*s. Concurrently, negative regulators of auxin signaling (e.g., *VvIAA9* and *VvARF2*) maintained high expression levels during seed abortion. Based on this transcriptomic evidence, we propose that shifts in phytohormone homeostasis—specifically, surges in stress signals coupled with the suppression of growth-promoting signals—synergistically activate PCD and autophagy [50]. Consequently, PCD-related and *ATG* genes act as critical signal amplifiers, translating upstream hormonal shifts into irreversible cellular degradation. However, fully deciphering this biochemical crosstalk requires integrating targeted metabolomic analyses with functional characterization.

Fundamentally, this broad regulatory network is coordinated by TFs, which serve as central signaling hubs during organ development and degeneration [55]. Our spatiotemporal transcriptome analysis revealed that specific TF families (e.g., upregulated ERF, bHLH, and NAC; downregulated WRKY and TCP) are highly co-expressed with PCD-related and *ATG* genes during grape seed abortion. While extensive research confirms that TF-mediated regulation of PCD and autophagy is a conserved mechanism in plant reproductive development [50,56], with members such as ERF, bHLH, and WRKY directly targeting these pathways [55,57,58,59,60], our study provides direct functional evidence in grapevine. Crucially, we validated this regulatory network through *in vivo* transient fruit transformation assays. We demonstrated that both VvWRKY46 and VvERF71 upregulate *VvγVPE*. However, they exhibited precise functional divergence in their negative regulatory roles: VvWRKY46 inhibited *VvδVPE*, whereas VvERF71 suppressed *VvMC1.1*. This precise differential targeting reveals that these TFs do not act as binary switches for cell death. Rather, they fine-tune irreversible seed abortion at specific developmental nodes by balancing distinct PCD execution pathways (namely, the VPE and MC pathways). In summary, the dynamic expression of distinct TF families and their differential regulation of PCD and autophagy targets constitute the core molecular network underlying seed abortion. Ultimately, these identified core TFs and their targets provide valuable candidate genes for the precise manipulation of seedless traits via modern gene-editing technologies [61]. While this study establishes a macro-level regulatory network, future high-resolution or single-cell transcriptomics will be essential to overcome our broad sampling windows and better capture rapid transitional dynamics. Additionally, ongoing studies will focus on experimentally validating key promoter interactions and verifying the presence and dynamic accumulation of critical regulators at the protein level during seed abortion.

## 4. Materials and Methods

### 4.1. Plant Materials and Transcriptome Sequencing

The pseudo-parthenocarpic grapevine cultivar ‘Jingkejing’ was sourced from the National Grape Germplasm Repository (Zhengzhou Fruit Research Institute, Chinese Academy of Agricultural Sciences). Developing seeds of this cultivar were collected at three stages: 5–20 days after anthesis (DAA; young fruit stage, JY), characterized by normal early development; 23–31 DAA (seed-hardening stage, JS), marking the onset of seed abortion and coat hardening; and 34–46 DAA (abortion stage, JB), exhibiting complete seed abortion. These sampling windows accommodate the asynchronous development of seeds within clusters, ensuring representative sampling for each physiological state. Transcriptome sequencing (RNA-seq) was performed using the Illumina HiSeq™ 4000 (Illumina, San Diego, CA, USA) platform.

### 4.2. Identification of PCD-Related and ATG Gene Family Members in Grapevine

Genomic, protein, and annotation data for grapevine (*Vitis vinifera* L.) were retrieved from Ensembl Plants (https://plants.ensembl.org/index.html (accessed on 26 September 2024)). The amino acid sequences of the VPE, MC, and ATG families from The Arabidopsis Information Resource (TAIR) (https://www.arabidopsis.org/index.jsp (accessed on 28 September 2024)) were used as references. Local BLAST search were conducted in TBtools version 2.446 with an E-value threshold of <10^−5^ [62]. Redundant sequences sharing identical accession numbers were subsequently removed. Candidate sequences were analyzed for conserved domains using InterPro (https://www.ebi.ac.uk/interpro/ (accessed on 28 September 2024)) and NCBI CDD (https://blast.ncbi.nlm.nih.gov/ (accessed on 28 September 2024)) to identify PCD-related and ATG family members in grapevine. Target genes were named based on phylogenetic relationships. Distinct members clustering within the same subclade were distinguished using decimal suffixes (e.g., VvMC1.1) after confirming the absence of other paralogs.

### 4.3. Physicochemical Characterization and Subcellular Localization of PCD-Related and ATG Proteins in Grapevine

Physicochemical properties of candidate proteins, including theoretical isoelectric point (pI), molecular weight (MW), and sequence length, were calculated using ExPASy (https://www.expasy.org (accessed on 6 October 2024)). Subcellular localization was predicted using Cell-PLoc 2.0 (http://www.csbio.sjtu.edu.cn/bioinf/Cell-PLoc-2/ (accessed on 6 October 2024)) [63].

### 4.4. Chromosomal Localization of the PCD-Related and ATG Gene Families in Grapevine

Chromosomal locations of these genes were mapped using TBtools. Final graphical adjustments were performed using Adobe Illustrator version 2026.

### 4.5. Gene Structure Analysis of the PCD-Related and ATG Genes in Grapevine

The conserved domains of the encoded proteins were predicted using the NCBI Conserved Domain Database (CDD). Exon–intron structures and conserved domains were visualized using TBtools.

### 4.6. Phylogenetic Analysis of the PCD-Related and ATG Gene Families

Multiple sequence alignments of PCD-related and ATG proteins from *Vitis vinifera* and *Arabidopsis thaliana* were performed using MEGA 11. A phylogenetic tree was constructed using the neighbor-joining (NJ) method with the p-distance model, pairwise deletion, and 1000 bootstrap replicates. Tree visualization and annotation were performed using the iTOL web server (https://itol.embl.de/ (accessed on 10 October 2024)) [64].

### 4.7. Intraspecific and Interspecific Collinearity Analysis of PCD-Related and ATG Genes

Grapevine chromosome lengths and target gene coordinates were extracted using TBtools. Intraspecific collinearity analysis was conducted using MCScanX implemented in TBtools, and the resulting syntenic relationships were visualized as Circos plots.

For interspecific collinearity analysis, pairwise comparisons between grapevine and *Arabidopsis thaliana*, *Nicotiana tabacum*, *Zea mays*, and *Solanum lycopersicum* were conducted using MCScanX in TBtools. A multi-species collinearity map was constructed using TBtools, with final graphical adjustments performed in Adobe Illustrator.

### 4.8. Differential Expression Analysis of PCD-Related and ATG Gene Families in Grapevine

The FPKM (Fragments Per Kilobase of transcript per Million mapped reads) values for the PCD-related and *ATG* gene families across three developmental stages (JY, JS, and JB) were extracted from the transcriptome datasets. To systematically identify the differentially expressed genes (DEGs) across the critical developmental transitions (JY vs. JS, JS vs. JB, and JY vs. JB), the identification criteria were strictly set to a fold-change threshold of |log2FC| ≥ 1 and a False Discovery Rate (FDR) of <0.05. The FDR was calculated using the Benjamini–Hochberg correction method for multiple testing. A gene expression heatmap with hierarchical clustering was generated using TBtools [62].

### 4.9. Analysis of Promoter Cis-Acting Elements and Potential Transcription Factors

Promoter sequences (2 kb upstream of the translation start sites) of target genes were extracted using TBtools. These sequences were analyzed using PlantCARE (https://bioinformatics.psb.ugent.be/webtools/plantcare/html/ (accessed on 3 October 2024)) to identify *cis*-acting regulatory elements, and visualized using TBtools. Putative transcription factors targeting these promoters were predicted using PlantRegMap (https://plantregmap.gao-lab.org/ (accessed on 1 March 2026)).

### 4.10. Protein–Protein Interaction Analysis of PCD-Related and ATG Proteins

The amino acid sequences of grapevine VPE, metacaspase, and ATG proteins were queried against the STRING (https://string-db.org/ (accessed on 6 October 2024)) for protein–protein interaction (PPI) analysis. The interaction confidence threshold was set to a score of 0.400 (medium confidence). Resulting data were imported into Cytoscape version 3.9.1 to construct and visualize the PPI network.

### 4.11. Agrobacterium-Mediated Transient Transformation

*Agrobacterium tumefaciens* strain GV3101 harboring the recombinant plasmids was resuspended in an infiltration buffer (10 mM MES, 10 mM MgCl_2_, pH 5.6). The bacterial suspension was adjusted to an OD_600_ of 1.0, and acetosyringone was added to a final concentration of 100 µM prior to infiltration. Berries were injected for *Agrobacterium*-mediated transient expression according to a previously described protocol [65]. After infiltration, genomic DNA and total RNA were extracted from infiltrated berries using a modified CTAB method [66]. To identify positive transformants, PCR was performed on genomic DNA using vector-specific primers (Table S2), followed by agarose gel electrophoresis. Only total RNA extracted from the PCR-positive berry samples was subsequently reverse-transcribed into cDNA. Quantitative real-time PCR (qRT-PCR) was performed to analyze target gene expression (Table S3). All experiments included three biological replicates per sample, and the relative expression levels were calculated using the 2^−ΔΔCt^ method [67].

## 5. Conclusions

In summary, this study identified nine core PCD-related genes (three *VPE* and six *MC* genes) and 33 *ATG* genes, associated with grape seed abortion, elucidating their potential molecular regulatory mechanisms. Spatiotemporal expression profiling revealed that multiple *VPE*, *MC*, and *ATG* genes, particularly *VvγVPE* and *VvMC9*, exhibited specific expression patterns, highlighting their crucial roles in this process. Concurrently, five major phytohormone pathways (ABA, GA, IAA, JA, and SA) displayed distinct response profiles during seed abortion, with a potential interaction identified between an IAA pathway-associated flavin monooxygenase and MC proteins. Moreover, transcription factors from the ERF, WRKY, bHLH, and NAC families were significantly differentially expressed, implicating them as upstream regulators of PCD-related and *ATG* genes. Functional assays confirmed the regulatory specificity of the core transcription factors VvWRKY46 and VvERF71: both activate *VvγVPE* expression, whereas VvWRKY46 represses *VvδVPE* and VvERF71 represses *VvMC1.1*. These findings demonstrate that transcription factors coordinate the seed abortion program through the differential regulation of distinct PCD pathways. Collectively, this work provides a theoretical foundation for understanding seedless grape formation and identifies valuable candidate genes for molecular marker-assisted breeding and genetic engineering of seedless traits.

## Figures and Tables

**Table 1:** *Ka*/*Ks* analysis of collinear gene pairs within the *ATG* gene family in grapevine.

Collinear Gene Pair	*Ka*	*Ks*	*Ka*/*Ks*
*VvATG8c.1-VvATG8a*	0.126	0.582	0.216
*VvATG8c.1-VvATG8c.2*	0.099	1.008	0.098
*VvATG8a-VvATG8c.2*	0.041	0.780	0.526
*VvATG18h-VvATG18g*	0.234	0.908	0.258
*VvATG8f.2-VvATG8f.1*	0.021	0.896	0.023

*Ka*, non-synonymous substitution rate. *Ks*, synonymous substitution rate. A *Ka*/*Ks* ratio < 1 indicates purifying selection, *Ka*/*Ks* = 1 indicates neutral evolution, and *Ka*/*Ks* > 1 indicates positive selection.

## Data Availability

All data generated or analyzed during this study are included in this published article and its Supplementary Materials. The raw RNA-seq data supporting this study are publicly available in the NCBI under the accession number PRJNA1156312.

## Supplementary Materials

The following supporting information can be downloaded at: https://www.mdpi.com/article/10.3390/ijms27188353/s1.
