# Supplementary material for: A Regulatory Network of Transcription and Hormones Controls PCD and Autophagy in Grape Seed Abortion
Source: Int J Mol Sci. 2026 Sep 19;27(18):8353. doi: 10.3390/ijms27188353 (PMC13607523; doi:10.3390/ijms27188353)
Supplement: Supplementary file 1 [file ijms-27-08353-s001.zip › Figure S1.pdf]

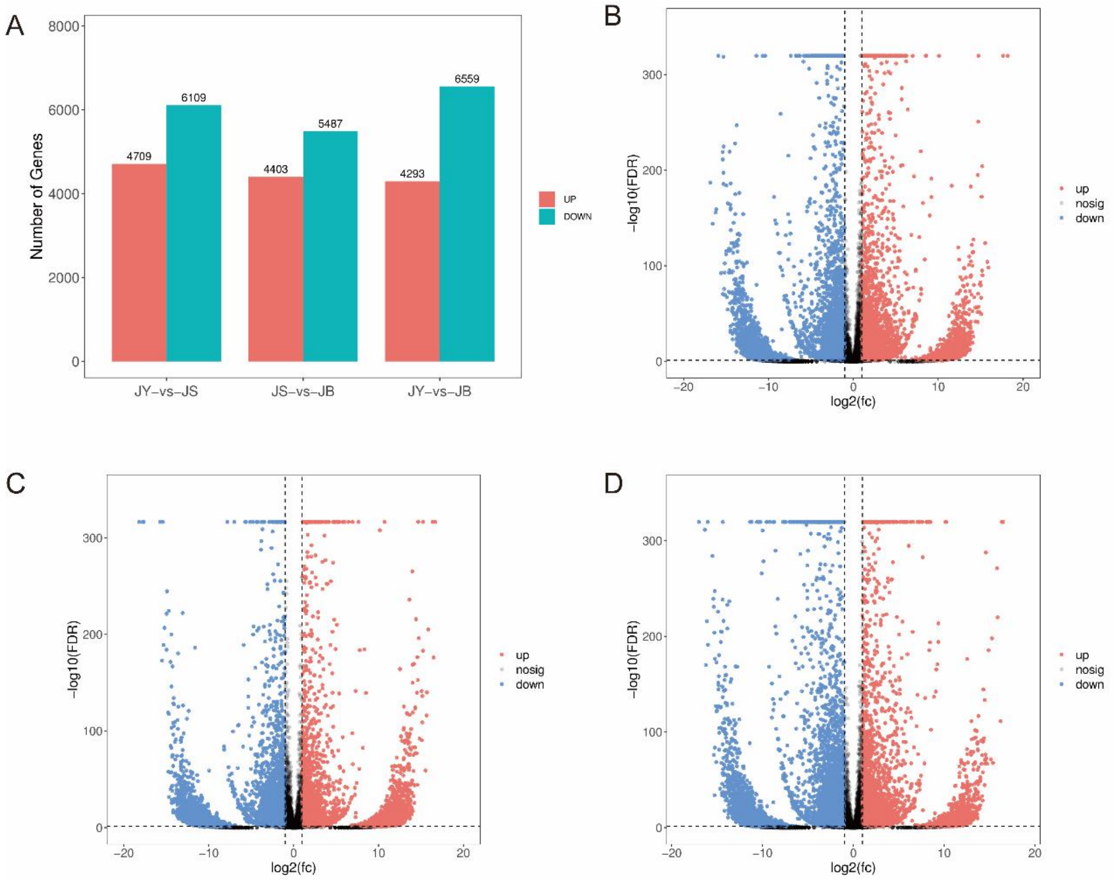

Figure S1. Global analysis of differentially expressed genes (DEGs) across different stages of grape seed abortion. (A) Statistical summary of the number of up-regulated (red bars) and down-regulated (green bars) DEGs in the JY vs. JS, JS vs. JB, and JY vs. JB comparisons. (B–D) Volcano plots illustrating the distribution of global gene expression in the JY vs. JS (B), JS vs. JB (C), and JY vs. JB (D) comparison groups. The x-axis indicates the log2(fold change) in gene expression, and the y-axis indicates the statistical significance plotted as -log10(FDR). Significantly up-regulated and down-regulated genes are highlighted in red and blue, respectively, whereas non-significantly expressed genes are shown in black. DEGs were identified using the thresholds of $|\log_2(fc)| \geq 1$ and FDR < 0.05.
