# Supplementary material for: A Regulatory Network of Transcription and Hormones Controls PCD and Autophagy in Grape Seed Abortion
Source: Int J Mol Sci. 2026 Sep 19;27(18):8353. doi: 10.3390/ijms27188353 (PMC13607523; doi:10.3390/ijms27188353)
Supplement: Supplementary file 1 [file ijms-27-08353-s001.zip › Table S1 .pdf]

Table S1 Physicochemical properties of grape VPE, MC, and ATG proteins

Gene name	Gene ID	Chromosome	Length	No. of aa	Mw(kDa)	PI	Predicted location(s)
VvβVPE	Vitvi02g00505	2	2712	494	55.44	5.37	Vacuole.
VvδVPE	Vitvi09g01380	9	3386	476	53.54	5.7	Vacuole.
VvγVPE	Vitvi04g04024	4	5702	493	54.03	5.28	Vacuole.
VvMC1	Vitvi15g00514	15	24135	370	39.98	6.62	Nucleus.
VvMC1.1	Vitvi16g00276	16	7723	362	39.42	6.41	Cell membrane. Chloroplast. Nucleus.
VvMC2	Vitvi16g00275	16	4449	359	40.33	6.66	Nucleus.
VvMC3	Vitvi03g01144	3	5654	361	40.76	8.75	Nucleus.
VvMC4	Vitvi19g02355	19	1370	424	46.25	5.05	Cytoplasm. Nucleus.
VvMC9	Vitvi08g00911	8	1056	316	34.88	5.89	Cytoplasm.
VvATG1a	Vitvi15g00869	15	7490	686	76.07	5.85	Nucleus.
VvATG1c.1	Vitvi01g01613	1	3016	466	52.12	8.74	Nucleus.
VvATG1c.2	Vitvi08g01267	8	9575	732	81.41	6.42	Nucleus.
VvATG2	Vitvi03g00492	3	27203	2020	221.63	5.27	Chloroplast. Nucleus.
VvATG3	Vitvi15g00604	15	21370	314	35.39	4.73	Nucleus.
VvATG4	Vitvi07g01236	7	8980	400	44.47	5.2	Cell membrane. Cytoplasm. Nucleus.
VvATG5	Vitvi02g00798	2	12735	265	30.11	5.85	Cytoplasm.
VvATG6	Vitvi15g00986	15	6136	522	59.3	5.68	Nucleus.
VvATG7	Vitvi10g00571	10	6624	787	86.68	5.79	Nucleus.
VvATG8a	Vitvi12g00370	12	3182	121	14.03	8.78	Cytoplasm. Nucleus.
VvATG8c.1	Vitvi10g04024	10	3656	177	20.36	9.3	Cytoplasm. Nucleus.
VvATG8c.2	Vitvi19g02021	19	3520	119	13.75	8.8	Cytoplasm. Golgi apparatus. Nucleus.
VvATG8f.1	Vitvi02g00535	2	4195	122	14.12	8.71	Cytoplasm. Golgi apparatus. Nucleus.
VvATG8f.2	Vitvi15g00718	15	6055	124	14.34	9.02	Cytoplasm. Nucleus.
VvATG8i	Vitvi07g00485	7	3370	129	15	5.8	Cytoplasm.
VvATG9	Vitvi07g00580	7	13075	958	111.37	6.23	Mitochondrion.
VvATG10	Vitvi07g02467	7	6327	230	26.49	5.16	Cytoplasm.
VvATG11	Vitvi11g00777	11	18533	1158	130.59	5.62	Nucleus.

Gene name	Gene ID	Chromosome	Length	No. of aa	Mw(kDa)	PI	Predicted location(s)
VvATG12	Vitvi09g00111	9	1856	94	10.52	9.21	Cytoplasm.
VvATG13a	Vitvi16g01309	16	4727	624	69.09	8.4	Nucleus.
VvATG13b	Vitvi04g01617	4	7403	660	72.58	8.68	Nucleus.
VvATG14	Vitvi18g00348	18	15016	478	54.03	9.03	Nucleus.
VvATG16	Vitvi17g00971	17	6239	541	59.98	6.11	Nucleus.
VvATG18a.1	Vitvi07g00280	7	3009	386	42.31	7.19	Nucleus.
VvATG18a.2	Vitvi07g00282	7	4097	424	46.78	6.72	Nucleus.
VvATG18a.3	Vitvi14g00191	14	5898	425	46.66	6.37	Nucleus.
VvATG18b	Vitvi11g00813	11	39593	368	40.13	8.25	Nucleus.
VvATG18c	Vitvi13g00009	13	2525	417	46.05	7.67	Nucleus.
VvATG18f	Vitvi19g01985	19	10665	922	99.31	7.15	Chloroplast.
VvATG18g	Vitvi07g00210	7	8035	988	107.73	6.05	Nucleus.
VvATG18h	Vitvi14g00317	14	9873	986	106.85	5.63	Nucleus.
VvATG36	Vitvi17g00440	17	5572	648	72.02	8.15	Nucleus.
VvATG101	Vitvi07g01302	7	6758	218	25.27	6.51	Chloroplast.
