# Supplementary material for: A Regulatory Network of Transcription and Hormones Controls PCD and Autophagy in Grape Seed Abortion
Source: Int J Mol Sci. 2026 Sep 19;27(18):8353. doi: 10.3390/ijms27188353 (PMC13607523; doi:10.3390/ijms27188353)
Supplement: Supplementary file 1 [file ijms-27-08353-s001.zip › Table S2 .pdf]

Table S2 Primer sequences for transient expression vector construction and PCR verification

Primer Name	Forward (5' - 3')	Use
VvERF71-1300-F	ggtcgacatttaaatactagtATGTGTGGAGGTGCTATCATCTC	Vector construction
VvERF71-1300-R	gcccttgctcaccatggtaccTAGAGCAGCTGAGGTGGGTT	Vector construction
VvWRKY46-1300-F	ggtcgacatttaaatactagtATGGAAATGGCCAGGGAGT	Vector construction
VvWRKY46-1300-R	gcccttgctcaccatggtaccTTGGAAGAATTCAGGATTATCAAAT	Vector construction
1300-F	CTCGATACACCAAATCGACTCTAG	PCR verification
1300-R	CACGCTGAACTTGTGGCCGTTT	PCR verification

The accession numbers for VvERF71 and VvWRKY46 are XM_002272428.4 and XM_002280995.4, respectively. Lowercase letters indicate homologous arms.
