# Supplementary material for: A Regulatory Network of Transcription and Hormones Controls PCD and Autophagy in Grape Seed Abortion
Source: Int J Mol Sci. 2026 Sep 19;27(18):8353. doi: 10.3390/ijms27188353 (PMC13607523; doi:10.3390/ijms27188353)
Supplement: Supplementary file 1 [file ijms-27-08353-s001.zip › Table S3 .pdf]

Table S3. Primer sequences for qRT-PCR

Primer Name	Forward (5' - 3')
VvγVPE-F	AACCCTAGGCCTGGAATCAT
VvγVPE-R	TGTCCAGAACCTTCCCACTA
VvδVPE-F	ATACAGTGTTGCCTGGTTGG
VvδVPE-R	GTGCCCATGTAGGTGAAGAG
VvMC1.1-F	TACTCGTCTACCCAAAGCCA
VvMC1.1-R	TAAGCATGAGAACGGAAGCC
VvMC9-F	ACTTCCGCAGACATGAATCC
VvMC9-R	TTTAGGCTGCCACAGGAAAG
VvATG8i-F	CCGGTGATTGCTGAAAGGTA
VvATG8i-R	TACAGGAACCCGTCCTCATC
VvATG101-F	ACACCCAAAAGCGCATTCTA
VvATG101-R	GGAAATGAGACACCCTCTGC
VvActin-F	GCTCGCTGTTTTGCAGTTCTAC
VvActin-R	AACATAGGTGAGGCCGCACTT
