# Supplementary material for: A Regulatory Network of Transcription and Hormones Controls PCD and Autophagy in Grape Seed Abortion
Source: Int J Mol Sci. 2026 Sep 19;27(18):8353. doi: 10.3390/ijms27188353 (PMC13607523; doi:10.3390/ijms27188353)
Supplement: Supplementary file 1 [file ijms-27-08353-s001.zip › Table S4.pdf]

Table S4 Differential expression status of PCD-related and *ATG* genes across different seed developmental stages.

Gene	Gene ID	JY vs. JS	JS vs. JB	JY vs. JB
VvβVPE	VIT_200s0323g00100	No	No	No
VvδVPE	VIT_209s0054g00690	Yes	Yes	No
VvγVPE	VIT_204s0008g00720	No	No	No
VvMC1	VIT_215s0021g00990	Yes	No	No
VvMC1.1	VIT_216s0013g00220	Yes	No	No
VvMC2	VIT_216s0013g00210	No	No	No
VvMC3	VIT_203s0017g01010	Yes	No	No
VvMC4	VIT_219s0085g01030	Yes	No	No
VvMC9	VIT_208s0040g00490	Yes	Yes	No
VvATG1a	VIT_215s0048g02400	No	No	No
VvATG1c.1	VIT_201s0010g03270	Yes	Yes	Yes
VvATG1c.2	VIT_208s0007g01150	No	No	No
VvATG2	VIT_203s0063g00600	No	No	No
VvATG3	VIT_215s0021g02130	No	No	No
VvATG4	VIT_200s0215g00030	No	No	No
VvATG5	VIT_202s0012g02270	No	No	No
VvATG6	VIT_215s0046g00950	No	Yes	No
VvATG7	VIT_210s0003g01150	Yes	Yes	No
VvATG8a	VIT_212s0059g00660	No	No	No
VvATG8c.1	VIT_210s0116g01700	Yes	No	No
VvATG8c.2	VIT_219s0090g00580	Yes	No	No
VvATG8f.1	VIT_202s0154g00390	Yes	Yes	Yes
VvATG8f.2	VIT_215s0048g00660	No	Yes	Yes
VvATG8i	VIT_207s0005g02200	No	No	No
VvATG9	VIT_207s0005g03100	No	No	No
VvATG10	VIT_207s0151g00710	Yes	Yes	No
VvATG11	VIT_211s0037g00730	No	No	No
VvATG12	VIT_209s0002g01390	No	No	No
VvATG13a	VIT_216s0098g00590	No	No	No
VvATG13b	VIT_204s0044g00610	No	No	No
VvATG14	VIT_218s0001g04540	No	No	No
VvATG16	VIT_217s0000g09750	No	No	No
VvATG18a.1	VIT_207s0104g01780	No	Yes	No
VvATG18a.2	VIT_207s0104g01790	No	Yes	No
VvATG18a.3	VIT_214s0060g02380	No	No	No
VvATG18b	VIT_211s0037g00970	Yes	Yes	No
VvATG18c	VIT_213s0067g00170	Yes	Yes	Yes
VvATG18f	VIT_219s0014g05190	No	No	No
VvATG18g	VIT_207s0104g00860	No	No	No
VvATG18h	VIT_214s0030g00400	No	No	No
VvATG36	VIT_217s0000g04770	Yes	No	No
VvATG101	VIT_200s0253g00070	No	Yes	No

Note: "Yes" indicates that the gene was identified as a significantly differentially expressed gene (DEG) in the corresponding comparison group, whereas "No" indicates no significant differential expression.
